# Supplementary material for: Relevant Criteria for Improving Quality of Schizophrenia Spectrum Disorders Treatment: A Delphi Study
Source: Healthcare (Basel). 2025 Nov 10;13(22):2847. doi: 10.3390/healthcare13222847 (PMC12652895; doi:10.3390/healthcare13222847)
Supplement: Supplementary file 1 [file healthcare-13-02847-s001.zip › Supplementary file S2.pdf]

Supplementary file S2. List of associations involved in focus groups

Associations involved in patient and informal caregivers' recruitment for the focus group:

- **Asociación Española de Apoyo en Psicosis (AMAFE)** (Spanish Association for Support in Psychosis)
- **Asociación Aragonesa Pro Salud Mental (ASAPME)** (Aragonese Association for Mental Health)

Scientific societies involved in the professional focus group:

- **Asociación Española de Apoyo en Psicosis (AMAFE)** *(Spanish Association for Support in Psychosis)*
- **Asociación Española de Enfermería de Salud Mental (AEESME)** *(Spanish Association of Mental Health Nursing)*
- **Sociedad Española de Directivos de la Salud (SEDISA)** *(Spanish Society of Health Managers)*
- **Sociedad Española de Farmacia Hospitalaria (SEFH)** *(Spanish Society of Hospital Pharmacy)*
- **Sociedad Española de Medicina de Familia y Comunitaria (semFYC)** *(Spanish Society of Family and Community Medicine)*
- **Sociedad Española de Médicos de Atención Primaria (SEMERGEN)** *(Spanish Society of Primary Care Physicians)*
- **Sociedad Española de Patología Dual (SEPD)** *(Spanish Society of Dual Pathology; Note:* "Dual pathology" *refers to the comorbidity of mental disorders and addictions)*

- **Sociedad Española de Psiquiatría y Salud Mental (SEPSM)** *(Spanish Society of Psychiatry and Mental Health)*
