# Supplementary material for: Relevant Criteria for Improving Quality of Schizophrenia Spectrum Disorders Treatment: A Delphi Study
Source: Healthcare (Basel). 2025 Nov 10;13(22):2847. doi: 10.3390/healthcare13222847 (PMC12652895; doi:10.3390/healthcare13222847)
Supplement: Supplementary file 1 [file healthcare-13-02847-s001.zip › Supplementary file S3.pdf]

Supplementary file S3. Focus group guides for patients and professionals

This document contains the scripts used to guide the focus group discussions conducted with (a) patients diagnosed with Schizophrenia Spectrum Disorders (SSD) and caregivers, and (b) healthcare professionals from different care levels.

A. Patient Focus Group Guide

Title:
Journey of the Patient with Schizophrenia Through Mental Health Services – Focus Group

Date and Location:
Madrid, June 26, 2023

Duration:
Approximately 2 hours

General Objective

To explore the experiences of patients with schizophrenia throughout their journey across mental health services, identifying perceived barriers, facilitators, and areas for improvement at the different levels of care.

Session Structure

1. Introduction (15 min)

- Welcome and explanation of the study's purpose.
- Reminder of confidentiality and voluntary participation.
- Brief introduction of participants.

2. Patient Journey and Care Received (90 min)

Participants were invited to reflect on their experiences across the different healthcare settings.

Guiding Questions:

- Primary Care: What barriers have you encountered in primary care?
- What positive aspects stood out to you and were helpful?
- Emergency Services: What barriers have you encountered in emergency care?
- What positive aspects stood out to you and were helpful?
- Hospital: What barriers have you encountered during hospital care or hospitalization (if applicable)?

- What positive aspects stood out to you and were helpful?
- Mental Health Unit (MHU): What barriers have you encountered in the Mental Health Unit?
- What positive aspects stood out to you and were helpful?
- Day Hospital: What barriers have you encountered in day hospital care?
- What positive aspects stood out to you and were helpful?
- Have you found any significant difficulties in other services? What were they?
- And what positive aspects in those services helped you?
- Other services: Throughout your entire journey across mental health services, is there any particular aspect you think could have improved your experience?

4. Closing (15 min)

- Summary of the main topics identified.
- Appreciation and reminder of the study's objective.

Note: All participants signed an informed consent form prior to the session, in accordance with the approval of the Ethics Committee of San Juan de Alicante University Hospital (Ref: 23/074).

B. Professionals Focus Group Guide

Title:

Definition of Quality Criteria for the Care of People with Severe Mental Disorders – Focus Group

Date and Location:

Madrid, July 10, 2023

Duration:

Approximately 2 hours

General Objective:

To identify improvement actions and define quality criteria for the care of people with severe mental disorders, based on the barriers and needs identified in the patient focus group.

Session Structure

1. Introduction (10 min)

- Welcome and presentation of the project objectives.
- Reminder of confidentiality and informed consent.
- Brief introduction of participants.

2. Presentation of Results from the Patient Focus Group (15 min)

- Presentation of the main critical points identified by patients:
    - Delay in diagnosis and lack of detection in primary care; insufficient knowledge of the disorder among professionals.
    - Insufficient specialized consultations to establish an adequate diagnosis.
    - Delays in follow-up visits or absence of scheduled appointments.
    - Repetitive and protocolized consultations with little patient-centered focus.
    - Need to improve healthcare professionals' communication skills and expand the information provided to patients about their illness, treatment process, and care.
    - Need to strengthen training for family members and caregivers to better support patients, and to provide greater support to families.
    - Lack of generalized support and deficiencies in addressing associated psychosocial problems.
    - Need for comprehensive care and improved coordination between services.
    - Gaps in the transition between the healthcare system and daily life during the stabilization phase.
    - Deficiencies in continuity of care and transitions between levels, particularly between child-adolescent and adult services.
    - Delay in identifying the appropriate pharmacological treatment.
    - Need to improve accessibility to healthcare resources, including psychiatry, psychology, nutrition, and sexology.
    - Need to optimize inpatient care for patients with Schizophrenia Spectrum Disorders (SSD), promoting the humanization of hospitalization processes.
    - Need to strengthen emergency care for patients with SSD.
    - Need to improve ambulance and patient transport services for individuals with SSD.
    - Stigma, lack of social awareness, and limited opportunities for individuals with the disorder to have their voices heard.

3. Discussion with Professionals (70 min)

Based on the results from the patient focus group, a structured discussion was conducted around the following guiding questions:

- What improvements are needed in the coordination between primary care, emergency services, hospitals, and mental health units?
- What aspects would facilitate more patient-centered care?
- Which quality indicators should be incorporated to evaluate care for patients with severe mental disorders (SMD)?
- What good practices or experiences would you highlight from your centers?

4. Synthesis and Prioritization (20 min)

- Summary of the emerging proposals.
- Joint identification of the most relevant and feasible quality criteria.

5. Closing (5 min)

- Appreciation to participants and reminder of the importance of their contribution to improving the quality of care.

Note: All participants signed an informed consent form prior to the session, in accordance with the approval of the Ethics Committee of San Juan de Alicante University Hospital (Ref: 23/074).
