# Supplementary material for: Relevant Criteria for Improving Quality of Schizophrenia Spectrum Disorders Treatment: A Delphi Study
Source: Healthcare (Basel). 2025 Nov 10;13(22):2847. doi: 10.3390/healthcare13222847 (PMC12652895; doi:10.3390/healthcare13222847)
Supplement: Supplementary file 1 [file healthcare-13-02847-s001.zip › Supplementary file S4.pdf]

Supplementary file S4. Delphi Round 1 questionnaire

This document contains the questionnaire used in Round 1 of the Delphi study on quality criteria for the care of people with Schizophrenia Spectrum Disorders (SSD).

Title:
Quality Criteria for the Care of People with Severe Mental Disorder (Schizophrenia).

Questionnaire Structure

The questionnaire was administered through an electronic form in two successive rounds. In Round 1, 46 criteria were included, grouped into 16 thematic dimensions, along with two additional items related to perceived outcomes (PROMs and PREMs).

Each criterion was rated using a Likert scale from 1 to 10, where:

- **1 =** Strongly disagree
- **10 =** Strongly agree

Participants were able to add open-ended comments to justify their ratings or suggest adjustments.

List of Delphi Round 1 Criteria (translated version)

Criterion 1.1. Provide school nursing services or, through family and community nurses trained in mental health, conduct educational campaigns in school settings.

Criterion 1.2. Provide training in Primary Care on psychosis in general and schizophrenia in particular to promote early detection and appropriate patient care.

Criterion 1.3. Have a Primary Care protocol that includes criteria for early detection (prodromal symptoms) and referral to Psychiatry.

Criterion 1.4. Provide a consultation channel with the reference Mental Health specialist for patients with Severe Mental Disorder (SMD, schizophrenia).

Criterion 2.1. Establish a communication channel between Primary Care and Psychiatry for discussing complex or uncertain cases.

Criterion 2.2. Ensure the availability of multidisciplinary teams for the detection and treatment of the disorder.

Criterion 2.3. Provide priority appointments with Psychiatry for patients with suspected SMD.

Criterion 3.1. Develop a follow-up protocol for stable patients with schizophrenia specifying the frequency of follow-up visits.

Criterion 3.2. Ensure early follow-up in the Psychiatry clinic after the first episode of schizophrenia (≤ 15 days).

Criterion 3.3. Provide access to consultation outside of routine follow-up visits in situations of risk of relapse.

Criterion 4.1. Conduct training and awareness activities for healthcare professionals to humanize care for patients with SMD (schizophrenia).

Criterion 4.2. Formulate an individualized treatment plan and therapeutic alliance. Note: The individualized plan and therapeutic alliance should be recorded in the patient's medical record.

Criterion 4.3. Assign a case coordinator (nurse, psychologist, or social worker) for each patient with SMD (schizophrenia) to coordinate their individualized rehabilitation plan.

Criterion 5.1. Provide communication skills training for professionals, including individualized information for each patient.

Criterion 6.1. Offer family psychoeducational therapy to prevent relapses and improve the prognosis of the disorder.

Criterion 6.2. Inform patients with SMD (schizophrenia) and their families about available social and healthcare support resources.

Criterion 6.3. Conduct training and educational workshops for patients with SMD (schizophrenia) and their families in collaboration with patient associations.

Criterion 6.4. Establish a communication channel between the Mental Health Center and patient associations.

Criterion 7.1. Ensure that patients with SMD (schizophrenia) receive psychosocial care from the multidisciplinary team.

Criterion 7.2. Implement peer-support programs as an integral part of the treatment plan for people with SMD (schizophrenia).

Criterion 8.1. Ensure that patients with SMD (schizophrenia) are treated in a multidisciplinary unit.

Criterion 8.2. Conduct joint follow-up meetings between Primary and Specialized Care and with addiction service networks.

Criterion 8.3. Provide an electronic medical record accessible to all services and levels of care involved in the management of patients with SMD (schizophrenia).

Criterion 9.1. Provide training for patients with SMD (schizophrenia) who require it in social skills and activities of daily living.

Criterion 9.2. Promote collaborative work between the healthcare team and patient associations for SMD (schizophrenia).

Criterion 10.1. Establish a care protocol to ensure a coordinated and appropriate transition from child-adolescent to adult mental health services.

Criterion 10.2. Provide intensive case management and community support services for early-onset patients with SMD (schizophrenia).

Criterion 11.1. Develop a clinical guideline specifying pharmacological treatment for both acute and stabilization phases.

Criterion 11.2. Maintain disease stability by avoiding hospital admissions whenever possible.

Criterion 11.3. Implement a system to evaluate patient safety.

Criterion 12.1. Develop a map of available resources in each healthcare area.

Criterion 12.2. Determine population-based resource needs according to the prevalence of schizophrenia.

Criterion 12.3. Establish a Social and Healthcare Network for Severe Mental Illness. Note: This network brings together available resources and allows case-by-case assessments.

Criterion 13.1. Establish a protocol to determine when and how physical restraint should be applied to patients with SMD (schizophrenia).

Criterion 13.2. Monitor physical harm (e.g., fractures, falls) in patients with SMD (schizophrenia).

Criterion 13.3. Nursing assistants and support staff should conduct educational and structured activities with patients to promote integration, manual and motor skills, and social interaction during hospitalization.

Criterion 14.1. Establish a care protocol for the management of patients with SMD (schizophrenia).

Criterion 14.2. Avoid the simultaneous use of multiple antipsychotic medications.

Criterion 14.3. Provide a dedicated space in emergency departments for psychiatric patients that ensures privacy and quality of care for patients with SMD (schizophrenia).

Criterion 15.1. Conduct training and awareness activities for professionals involved in the transfer of patients with SMD (schizophrenia) to promote humane care.

Criterion 15.2. Establish a specific protocol for hospital transfers of patients with SMD (schizophrenia) in acute phases, including urgent or involuntary transfers.

Criterion 16.1. Develop a joint communication plan among the Mental Health Center, Patient Associations, and Hospital.

Criterion 16.2. Conduct awareness campaigns in collaboration with patients with SMD (schizophrenia), associations, and healthcare professionals.

Criterion 16.3. Promote media interventions by professionals to address mental illness in a professional manner and counter misinformation and false beliefs.

Criterion 1. Assess the quality of life of patients with SMD (Patient-Reported Outcome Measure, PROM).

Criterion 2. Implement a system to measure patient experience (Patient-Reported Experience Measure, PREM) in mental health services for people with SMD (schizophrenia).

Sociodemographic information of the panel

At the end of the questionnaire, the following data were collected:

- Sex
- Age
- Professional profile
- Autonomous Community (Province) where you work
- Years of experience in the care of patients with Severe Mental Disorder (SMD)
