# Supplementary material for: Relevant Criteria for Improving Quality of Schizophrenia Spectrum Disorders Treatment: A Delphi Study
Source: Healthcare (Basel). 2025 Nov 10;13(22):2847. doi: 10.3390/healthcare13222847 (PMC12652895; doi:10.3390/healthcare13222847)
Supplement: Supplementary file 1 [file healthcare-13-02847-s001.zip › Supplementary table S1.pdf]

Supplementary Table S1. Search strategy for literature review

The search strategy was conducted in May and June 2023 using the following databases: PubMed (Medline), ProQuest, and the International Journal of Integrated Care database. The search queries were structured using Boolean operators and relevant keywords to identify studies related to adherence, awareness of illness, integrated care, and patient journey in schizophrenia care. The final number of included articles was determined after removing duplicates.

Table S1. *Search Strategy for the Literature Review*

Topic	Database	Search Query	Results (N)
**Adherence**	PubMed (Medline)	((compliance[Title/Abstract]) AND (adherence[Title/Abstract])) AND (schizophrenia[Title/Abstract])	65
**Awareness of Illness**	PubMed (Medline)	((“lack of insight”[Title/Abstract]) OR (“illness unawareness”[Title/Abstract])) AND (schizophrenia[Title/Abstract])	46
**Integrated Care**	PubMed (Medline)	(schizophrenia[Title/Abstract]) AND (“integral care” OR “integrative care” OR “integrated care”)	49
	ProQuest	abstract(schizophrenia) AND abstract(“integral care” OR “integrative care” OR “integrated care”)	6
	Int. J. of Integrated Care	schizophrenia	4
	Duplicates removed		-1
	**Total**		**58**
**Patient Journey**	PubMed (Medline)	schizophrenia AND “patient journey”	5

ProQuest	abstract(schizophrenia) AND abstract("patient journey")	2
Duplicates removed		-2
**Total**		**5**
